# Identification of patients with suboptimal results after hip arthroplasty: development of a preliminary prediction algorithm

**DOI:** 10.1186/s12891-015-0720-1

**Published:** 2015-10-05

**Authors:** Eugen Lungu, Pascal-André Vendittoli, François Desmeules

**Affiliations:** Department of Biomedical Sciences, Faculty of Medicine, University of Montreal, Montreal, QC Canada; Centre de recherche de l’Hôpital Maisonneuve-Rosemont, 5415 Boul. L’Assomption, Montreal, Québec H1T 2M4 Canada; Department of Surgery, Faculty of Medicine, University of Montreal, Montreal, QC Canada; School of Rehabilitation, Faculty of Medicine, University of Montreal, Montreal, QC H3C 3J7 Canada

**Keywords:** Hip arthroplasty, Joint perception, Osteoarthritis, Prediction, Surgical outcomes

## Abstract

**Background:**

The ability to predict preoperatively the identity of patients undergoing hip arthroplasty at risk of suboptimal outcomes could help implement interventions targeted at improving surgical results. The objective was to develop a preliminary prediction algorithm (PA) allowing the identification of patients at risk of unsatisfactory outcomes one to two years following hip arthroplasty.

**Methods:**

Retrospective data on a cohort of 265 patients having undergone primary unilateral hip replacement (188 total arthroplasties and 77 resurfacing arthroplasties) from 2004 to 2010 were collected from our arthroplasty database. Hip pain and function, as measured by the Western Ontario and McMaster Universities Osteoarthritis Index (WOMAC) were collected, as well as self-reported hip joint perception after surgery. Demographic and clinical variables recorded at the time of the surgery were considered as potential predictors. Patients were considered as having a suboptimal surgical outcome if they were in the worst quartile of the postoperative total WOMAC score and perceived their operated hip as artificial with minimal or major limitations. The PA was developed using recursive partitioning.

**Results:**

Mean postoperative surgical follow-up was 446 ± 171 days. Forty patients (15.1 %) had a postoperative total WOMAC score in the worst quartile (>11.5/100) and perceived their joint as artificial with minimal or major restrictions. A PA consisting of the following variables achieved the most acceptable level of prediction: gender, age at the time of surgery, body mass index (BMI), and three items of the preoperative WOMAC (degree of pain with walking on a flat surface and during the night as well as degree of difficulty with putting socks or stockings). The rule had a sensitivity of 75.0 % (95 % CI: 59.8-85.8), a specificity of 77.8 % (95 % CI: 71.9–82.7), a positive predictive value of 37.5 % (95 % CI: 27.7–48.5), a negative predictive value of 94.6 % (95 % CI: 90.3–97.0) and positive and negative likelihood ratios of 3.38 (95 % CI: 2.49–4.57) and 0.34 (95 % CI: 0.19–0.55) respectively.

**Conclusions:**

The preliminary PA shows promising results at identifying patients at risk of significant functional limitations, increased pain and inadequate joint perception after hip arthroplasty. Clinical use should not be implemented before additional validation and refining.

**Electronic supplementary material:**

The online version of this article (doi:10.1186/s12891-015-0720-1) contains supplementary material, which is available to authorized users.

## Background

Recent recommendations suggest that total hip arthroplasty (THA) is indicated when the patients’ functional limitations and pain levels due to hip osteoarthritis (OA) are refractory to pharmacological and non-pharmacological treatments [1, 2]. Resurfacing hip arthroplasty (HR) is an alternative to THA in patients who are younger, more active, with normal renal function and appropriate proximal femoral bone morphology and quality [3]. Both THA and HR are considered efficacious for the great majority of patients undergoing these procedures [4–7]. Although generally successful at alleviating coxarthrosis-related ailments, hip arthroplasty can yield subpar results in terms of pain and functional outcomes as well as degree of satisfaction in a non-negligible proportion of patients. For example, a recent systematic review reports that 7 to 23 % of the patients undergoing THA experience unfavourable pain outcomes 3 months to 5 years after the procedure [8]. Moreover, up to 15 % of the patients report dissatisfaction with surgery [9, 10] . To our knowledge, no formal data on proportions of patients with poor pain, functional and satisfaction levels after HR exists. However, it can be posited that these proportions are similar to the ones observed among patients undergoing THA, as studies indicate that these outcomes are similar between the two procedures [11, 12].

In light of these observations, careful case management must be implemented in order to minimize unsuccessful outcomes. Potential interventions directed at improving surgical outcomes include patient education and intensive rehabilitation. However, identification of patients at risk of severe pain and functional limitations after THA or HR is difficult. A multitude of factors related to poor functional and pain outcomes following hip arthroplasty have been identified. These include worse preoperative levels of pain and function, lower educational level, comorbidities, presence of back pain or higher body mass index (BMI) among others [13–22]. Nevertheless, regardless of the quantity of the evidence of potential risk factors, no definitive consensus has been reached concerning their identity and the magnitude of their association with postoperative pain, functioning and satisfaction. In light these observations, an algorithm aimed at identifying with sufficient accuracy which patients present the greatest risk of unsuccessful outcomes may assist in the care process. Therefore, the objective of this study was to develop a preliminary prediction algorithm (PA) used to identify patients at risk of unfavourable functional status, pain and joint perception one to two years following THA or HR.

## Methods

### Study design

This study entailed a retrospective analysis of longitudinal, prospectively collected data. The methodology adheres to the Strengthening the Reporting of Observational Studies in Epidemiology (STROBE) guidelines for observational cohort studies (Additional file 1).

### Data collection

Our prospective arthroplasty database was consulted in order to identify patients eligible for inclusion in the study. The database contains extensive baseline and follow-up data on patients undergoing hip procedures, including THA and HR. All patients provide informed consent to participate. Independent assessors who are not involved in the medical care of the patients collect the prospective data.

Inclusion criteria were 1) patients undergoing primary unilateral THA or HR, 2) diagnosis of primary hip OA, 3) complete preoperative and one to two-year postoperative self-reported outcome questionnaire responses. The main exclusion criteria were 1) THA or HR of the contralateral hip before the relevant follow-up evaluation, 2) revision of the implant before the one to two-year follow-up, 3) diagnosis of inflammatory hip arthritis, pediatric hip disease, post-traumatic hip or any hip disease other than primary OA. Data on all patients having undergone hip interventions were assessed for inclusion. All patients were initially interviewed just before their intervention. Postoperative outcomes were collected 12 to 24 months after the surgery.

### Dependent variables

Functional status and pain levels were assessed preoperatively and at follow-up with the Western Ontario and McMaster Universities Osteoarthritis Index (WOMAC) [23]. The WOMAC consists of the following domains: pain (5 items), stiffness (2 items) and functional limitation (17 items). Items are scored on a 5-point Likert scale representing different degrees of intensity (none, mild, moderate, severe or extreme). The scores of each domain as well as the total score were standardized on a 0 to 100 scale, with a greater score indicating more pain, stiffness or functional limitation. The psychometric qualities of the WOMAC, including its responsiveness, convergent construct validity and reliability have been found excellent for evaluating patients with hip OA undergoing hip arthroplasty [24, 25].

At follow-up, self-perceived joint perception was measured by asking the patient a multiple-choice question: “How do you perceive your operated hip?” with the possible responses being ”Like a native or natural joint”, ”Like an artificial joint with no restriction”, ”Like an artificial joint with minimal restriction”, ”Like an artificial joint with major restriction” and ”Like a non-functional joint” [26]. Evaluation of joint perception has been strongly associated with validated clinical scores of patient-reported outcome measures and can be employed as a measure of patient satisfaction [26].

No consensus exists regarding what represents poor outcome following hip arthroplasty. Hence, patients of risk of suboptimal outcomes were defined as the ones in the worst quartile of the total WOMAC score at follow-up (i.e. WOMAC score >11.5) and perceiving their hip ”Like an artificial joint with minimal restriction”, "Like an artificial joint with major restriction” or "Like a non-functional joint”.

### Independent variables

#### Potential preoperative predictors

Several of the variables that were collected preoperatively and available in the database were considered as potential predictors of suboptimal hip arthroplasty outcome. Demographic variables included age and gender. Clinical variables included BMI, previous hip interventions and medical comorbidities (diabetes, gastrointestinal disease, immunosuppression secondary to corticosteroid use or other causes, cardiac disease, obesity, pulmonary disease, neurologic disease, urologic disease, and other comorbidities). Pain localization (back, radicular, buttocks, trochanter, groin, thigh, knee and/or calf) as well as whether hip pain was present at rest, after the first few steps, after a long walk and during sexual relations were also considered. Answers to the 24 individual items of the pre-operative WOMAC questionnaire were additionally included in the analysis as potential predictors.

### Statistical analysis

Baseline and follow-up mean WOMAC scores along with their standard deviations were calculated. Differences between time points in relation to total WOMAC scores and the respective domains was assessed using paired samples Student-t tests, with a significance level set at 0.05.

The classification and regression tree approach was used to build the PA as it is one of the most effective algorithms of recursive partitioning [27]. It is based on maximizing the within-node homogeneity by evaluating all combinations of potential predictors, thus minimizing the within-node error. The Gini impurity measure was used as a splitting criterion to develop the decision trees [28]. Data for all the patients in the training set was used to develop the PA. Firstly, all the potential predictor variables were employed to develop models using an automated approach. Secondly, a manual approach entailed the development of additional models by inputting independent variables that were judged to be more readily available and easier to employ in a clinical setting. For example, age and gender were favoured over the number of comorbidities and previous hip interventions because the latter two could be affected by a recall bias or would require extensive medical file review. The predictive values of every model were calculated along with their 95 % confidence intervals, namely sensitivity, specificity, positive and negative predictive values as well as positive and negative likelihood ratios [29]. Among all the proposed models, the one that showed the highest level of sensitivity and an acceptable level of specificity and that fit the ease-of-use criterion was selected in order to develop the screening tool. Internal validity of the model was then evaluated by the use of 1,000 bootstrap resamples [30]. All analyses were performed using IBM SPSS Statistics Version 20.0 (SPSS Inc., Chicago).

### Ethics

The research ethics committee of our centre approved the study annually.

## Results

### Participants

Our database yielded 2963 entries with at least some preoperative data on hip arthroplasty procedures performed from October 2004 to February 2014. Out of these, 1207 procedures (40.7 %) fit the inclusion criteria. Incomplete preoperative and/or postoperative data required for the purposes of the current study obliged the exclusion of a further 942 entries. Thus, a total of 265 primary hip arthroplasty interventions (60 classical THAs, 128 large-femoral head diameter THAs, and 77 h) with complete preoperative and postoperative data were included in the study (follow-up mean ± SD: 446.3 ± 171.1 days), representing a participation proportion of 22.0 %.

Table 1 shows selected characteristics of the 265 patients included in the study. The mean age of the participants was 52.0 (SD 9.0) and 67.4 % were male. The mean BMI was 28.2 (SD 5.1) and each patient had on average 0.79 comorbidities (SD 0.96).

**Table 1:** Selected characteristics of the participants who underwent hip arthroplasty (*n =* 265)

Variables considered for PA development	n (%)	Mean (SD)	Other collected variables	n (%)
Demographics			Contralateral hip status	
Age (years)		52 (9.0)	Unaffected	134 (50.4)
Female	89 (33.6)		Affected, not operated	105 (39.7)
Clinical characteristics			Unavailable	26 (9.9)
BMI ^¬^ (kg/m^2^)		28.2 (5.1)	Charnley class	
Medical comorbidities			Charnley A	124 (46.8)
Diabetes	19 (7.2)		Charnley B	87 (32.8)
Gastrointestinal disease	16 (6.0)		Charnley C	13 (4.9)
Immunosuppression	3 (3.0)		Unavailable	41 (15.5)
Cardiac disease	21 (7.9)		Employment status	
Obesity	41 (15.5)		Employed	163 (61.5)
Osteoporosis	2 (0.8)		Household	44 (16.6)
Pulmonary disease	15 (5.7)		Retired	9 (3.4)
Neurological disease	1 (0.4)		Other	11 (4.2)
Urological disease	1 (0.4)		Unavailable	38 (14.3)
Other	91 (34.3)		Walking aid	
None	124 (46.8)		Incapable with aid	5 (1.9)
Presence of back pain	40 (15.1)		Crutches	1 (0.4)
Pain localization			Two canes	31 (11.7)
Buttocks	128 (48.3)		Cane on a permanent basis, instability	118 (44.5)
Trochanter	164 (61.9)		Cane for outdoor activities	42 (15.8)
Groin	177 (66.8)		Cane for long distance walking	43 (16.2)
Thigh	124 (46.8)		Unavailable	25 (9.5)
Knee	111 (41.9)		Knee(s) status	
Calf	36 (13.6)		Affected	28 (10.5)
Radicular	6 (2.2)		Unaffected	204 (77.0)
Elsewhere	3 (1.1)		Unavailable	33 (12.5)
Presence of hip pain			Level of activity in the 3 months before surgery	
At rest	148 (55.8)		Heavy work/sport	26 (9.8)
After first few steps	182 (68.8)		Moderate work	53 (20.0)
After a long walk	224 (84.5)		Mild work/walking	112 (42.3)
During sexual intercourse	156 (58.9)		Sedentary	34 (12.8)
			Immobile	6 (2.3)
			Unavailable	34 (12.8)
			Duration of walking before eliciting pain	
			Walking unaffected	37 (14.0)
			31–60 min	51 (19.2)
			11–30 min	82 (30.9)
			2–10 min	53 (20.0)
			<2 min	16 (6.0)
			Walking impossible	1 (0.4)
			Unavailable	25 (9.5)

*SD* standard deviation, *BMI* body mass index

Mean follow-up was 446 (SD 171) days and ranged from 253 to 1638 days. Postoperatively, the patients had significantly improved on pain (−44.9, SD 22.6, 95 % CI −42.1 to −47.6), stiffness (−44.6, SD 25.1, 95 % CI −41.6 to −47.7), function (−43.6, SD 21.9, 95 % CI −40.9 to −46.2) as well as total WOMAC score (−43.9, SD 21.1, 95 % CI −41.4 to −46.5) (Table 2). Seventy-six patients (29 %) reported that they perceived their prosthetic joint as artificial with minimal or major restrictions (Table 3).

**Table 2:** Changes in WOMAC scores of the participants between preoperative measurement and following hip arthroplasty (*n =* 265)

	Mean preoperative score^a^ (SD)	Mean preoperative score^a^ (SD)	Change in score^b^ (SD)	95 % CI	Comparison between time points *(p* value)
WOMAC					
Pain	55.4 (19.2)	10.5 (16.7)	- 44.9 (22.6)	- 42.1 to–47.6	<0.001*
Stiffness	57.1 (19.4)	12.5 (18.1)	- 44.6 (25.1)	- 41.6 to–47.7	<0.001*
Function	53.2 (20.0)	9.6 (15.3)	- 43.6 (21.9)	- 40.9 to–46.2	<0.001*
Total score	54.0 (18.7)	10.1 (15.1)	- 43.9 (21.1)	- 41.4 to–46.5	<0.001*

*SD* standard deviation

*CI* confidence interval

^a^Scores presented as standardized scores. Lower scores sign a better condition. Scores were measured on the day of the surgery

^b^Negative changes in score indicate an improvement of the condition. Scores were measured on a mean of 446 ± 171 days following the intervention

*p < 0.05

**Table 3:** Postoperative joint perception of the patients who underwent hip arthroplasty according to the distribution of their postoperative total WOMAC scores (*n =* 265)

Joint perception						
WOMAC quartile	Native/Natural	Artificial with no restrictions	Artificial with minimal restrictions	Artificial with major restrictions	Non-functional	TOTAL
First	56	21	4	0	0	81
Second	32	9	6	0	0	47
Third	28	14	25	1	0	68
Fourth	17	12	35^a^	5^a^	0	69
TOTAL	133	56	70	6	0	265

^a^patients considered at risk of suboptimal outcome (*n =* 40); a higher quartile indicates a worse total WOMAC score at follow-up

Out of the 265 patients eligible for inclusion in the study, 40 (15.1 %) had a total WOMAC score > 11.5 and perceived their joint as artificial with minimal or major restrictions. Hence, these patients were considered as having suboptimal surgical outcomes.

### Final prediction algorithm

**Fig. 1:**
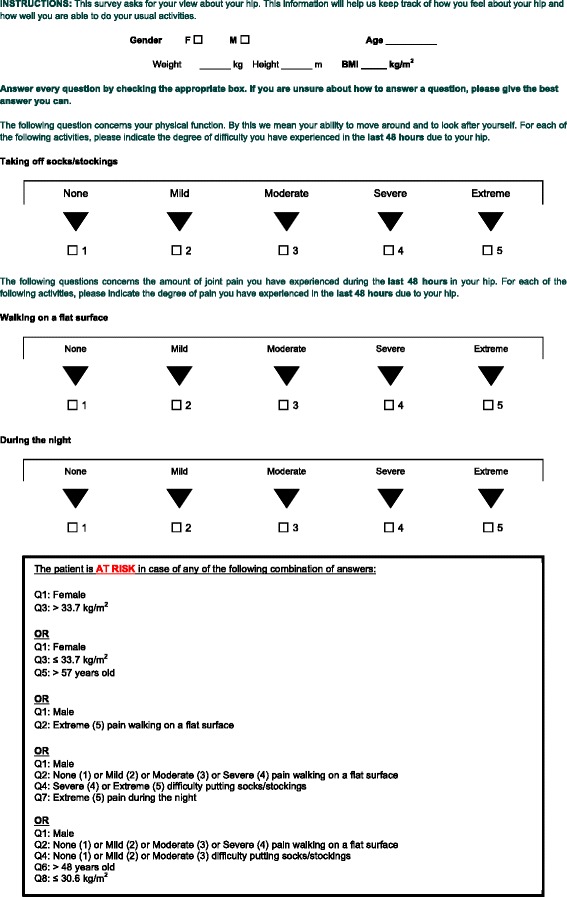
Prediction algorithm to identify patients at risk of suboptimal outcomes after hip arthroplasty

**Fig. 2:**
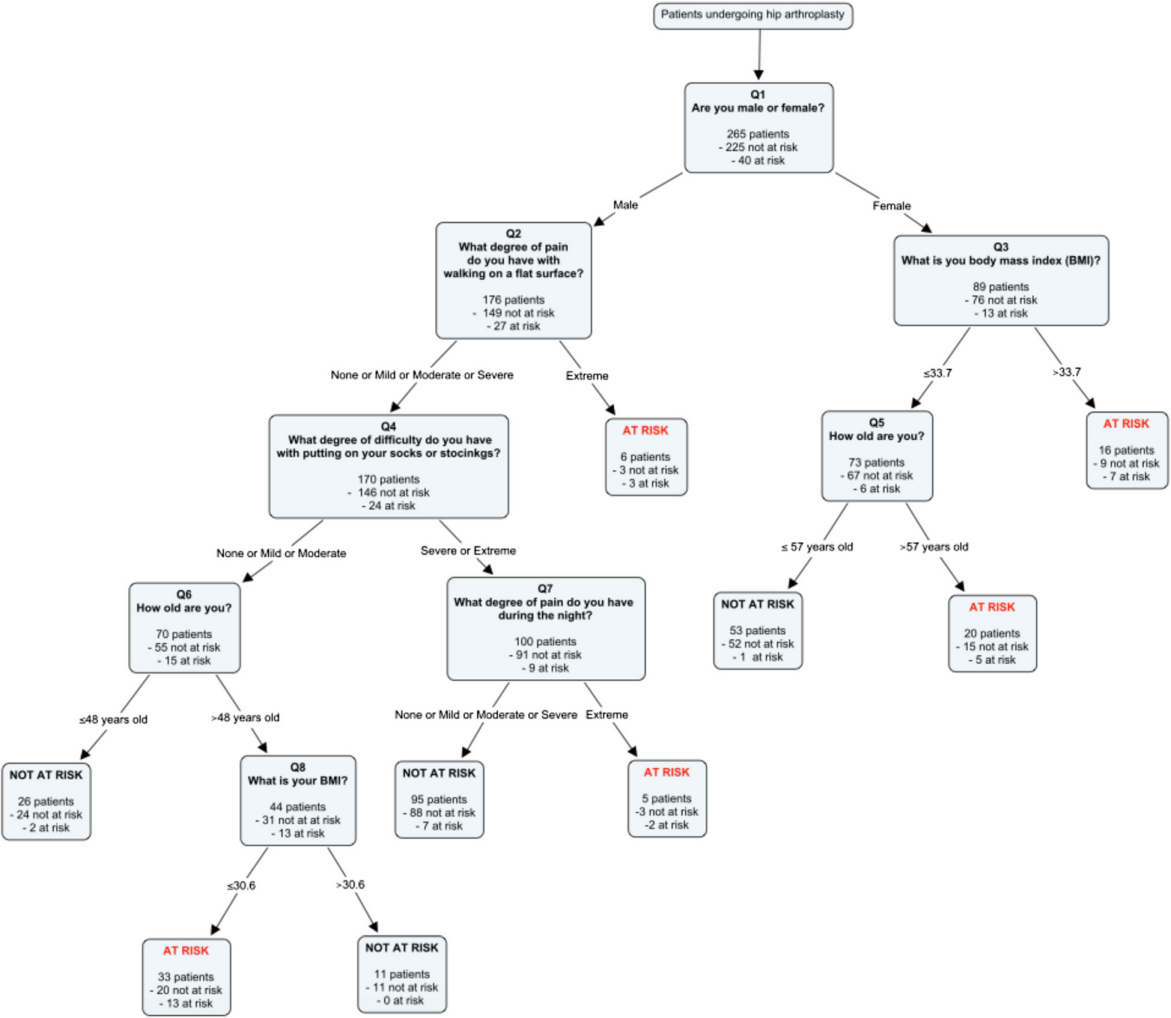
Graphical representation of the prediction algorithm identifying patients at risk of suboptimal surgical outcomes after hip arthroplasty

After developing several prediction rules, the algorithm with the highest level of sensitivity and an appropriate level of specificity was chosen. It consists of patient gender, age at the time of surgery, body mass index (BMI), and 3 items of the preoperative WOMAC, namely degree of pain with walking on a flat surface and during night and degree of difficulty with putting socks or stockings (Fig. 1). Patients respond sequentially to the questions and their risk status is determined according to the classification algorithm (Fig. 2).

The final PA correctly identified 30 out of the 40 patients considered at risk of suboptimal outcome and 175 patients out of 225 were identified as not at risk of suboptimal outcome (Table 4). Therefore, the PA had a sensitivity of 75.0 % (95 % CI: 59.8–85.8), a specificity of 77.8 % (95 % CI: 71.9–82.7 and a positive likelihood ratio of 3.38 (95 % CI: 2.49–4.57) (Table 5). The other prediction models that were also considered are presented in Additional file 2.

**Table 4:** Two by Two table of predicted versus actual outcomes of the prediction algorithm

	Actual outcome	
	AT RISK	NOT AT RISK
Predicted outcome	Worst postoperative WOMAC quartile (>11.5/100) & “Artificial with minimal or major limitations” joint perception	Postoperative WOMAC ≤ 11.5 &‘’Artificial with no limitations” or ‘’Natural joint” joint perception
AT RISK	30	50
NOT AT RISK	10	175
Total	40	225

**Table 5:** Validity measures of the prediction algorithm

Measure	Estimates in training sample	Estimates with 1,000 bootstrap resamples
Sensitivity % (95 % CI)	75.0 (59.8.4–85.8)	75.0 (60.0–88.0^a^)
Specificity % (95 % CI)	77.8 (71.9–82.7)	77.8 (72.2–82.9^a^)
Positive predictive value % (95 % CI)	37.5 (27.7–48.5)	37.2 (27.2–47.2^a^)
Negative predictive value % (95 % CI)	94.6 (90.3–97.0)	94.7 (91.2 to 97.8^a^)
Positive likelihood ratio (95 % CI)	3.38 (2.49–4.57)	3.38 (2.50 to 4.63^a^)
Negative likelihood ratio (95 % CI)	0.32 (0.19–0.55)	0.32 (0.15 to 0.52^a^)

• ^a^95 % asymptotic confidence intervals

• Sensitivity: number of participants classified at risk both by the PA and the postoperative WOMAC score and joint perception divided by all participants classified at risk by the postoperative WOMAC score and the joint perception (actual outcome)

• Specificity: number of participants classified not at risk by the PA and the postoperative WOMAC score and joint perception divided by all participants classified not at risk by the postoperative WOMAC score and joint perception (actual outcome)

• Positive predictive value: number of participants classified at risk by the PA and the post-operative WOMAC score and joint perception divided by all participants classified at risk by the PA (predicted outcome)

• Negative predictive value: number of participants classified not at risk by the PA and the postoperative WOMAC score and joint perception divided by all participants classified not at risk by the PA (predicted outcome)

• Positive likelihood ratio: sensitivity/ (1-specificity)

• Negative likelihood ratio: (1-sensitivity)/specificity

### Internal validation

Validation of the rule was established using 1,000 bootstrap re-samples. Table 5 indicates the estimated bootstrap values of the predictive measures being close to the original ones, thus suggesting an appropriate accuracy of the proposed model.

## Discussion

Since THA and HR can bring significant improvement in patients suffering from hip OA, careful management of subjects at risk of having unsuccessful outcomes is indicated. We aimed to develop a prediction tool in order to facilitate the preoperative identification of these patients, which could possibly ameliorate their surgical outcomes. With a cohort of 265 patients undergoing primary hip arthroplasty for OA, we were able to create a PA predicting the identity of patients that are at the highest risk of unsuccessful outcomes. Albeit preliminary in nature and requiring further development and validation, our PA has excellent predictive capacities, with a sensitivity of 75.0 % (95 % CI 59.8.4–85.8), a specificity of 77.8 % (95 % CI 71.9–82.7) and a positive likelihood ratio of 3.38 (95 % CI 2.49–4.57).

To our knowledge, one model predicting the identity of patients at risk of poor outcomes after THA has been developed [31]. Consisting of patient age, BMI and gender, the model was able to correctly predict patients’ outcomes with a sensitivity of 87.5 % (95 % CI 52.9–97.8), a specificity of 72.4 % (95 % CI 54.3–85.3) and a positive likelihood ratio of 3.17 (95 % CI 1.66–6.05). However, surgical success was determined solely based on functional outcomes (change between the preoperative and six-month Lower Extremity Functional Scale score), and the results are based on a cohort of 37 patients.

The selection of patients for inclusion in the study was based on the availability of complete data for important determinants of hip arthroplasty outcomes as reported in the literature. This allowed the development of a prediction rule that is consistent with the clinical reality. Because there is no unequivocal definition of what represents suboptimal outcome following hip replacement, several criteria of classifying patients who are at risk have been considered and different prediction models were built accordingly. The choice of the final model was based on the principle of selecting a screening tool minimizing the number of false negatives and that is easily employable in a clinical setting. Accordingly, an algorithm with a sensitivity of 75.0 % and a specificity of 77.8 % was deemed suitable. Although the positive likelihood ratio of 3.38 of the PA can be considered subpar when compared to accepted diagnostic standards, the PA performs similarly to other validated prediction models in the epidemiological literature. For example, the positive likelihood ratios of the Ottawa Knee and Ankle rules assessing the necessity of a roentgenographic evaluation in cases of acute knee and ankle injuries respectively are both inferior to the one reported by our PA [32, 33].

The algorithm with the most appropriate predictive capabilities contains two demographic variables (gender and age), one clinical (BMI) and 3 items of the preoperative WOMAC questionnaire (two pain-related and one function scale). All of these variables have been consistently related to hip arthroplasty outcomes [18–20, 22, 34, 35]. Moreover, the PA comprises all the predictors reported by Slaven et al. [31] in their model, namely age, gender and BMI, thus pointing towards the importance of these factors for prediction of hip arthroplasty results. It is noteworthy to mention that the prediction of surgical outcomes in women is achieved by age and BMI, with the body mass being the only modifiable risk factor. In the case of men, potential modifiable risk factors include BMI, degree of hip pain walking on a flat surface and during the night as well as degree of difficulty putting on socks or stockings. However, caution should be used, as recursive partitioning does not imply a causative relationship between variables [36]. Indeed, interventions targeted at ameliorating either of the items of the PA, such as weight loss in the case of high BMI, will not necessarily improve the outcome of the surgery; it will merely imply that the patient will be classified as not at risk of suboptimal outcomes by the PA. Further research in terms of appropriate interventions to improve surgical outcomes should be undertaken.

When developing the PA, we intended for it to be a clinically pertinent tool. The decision to include patients with different types of hip arthroplasties was taken in order to generate a PA that has the ability to perform successfully in a heterogeneous population. Moreover, we included subjects with complete information one to two years following the procedure, as patients are followed closely by their surgeons during this period, and the rehabilitation process can easily be altered if the progression is judged suboptimal.

In one instance, the interpretation process may yield a counterintuitive situation. For example, it is possible, in an extreme scenario, for a 49 year-old male patient with a BMI of 22 kg/m^2^ and with no pain when walking on a flat surface as well as with no difficulty with putting on socks or stockings to be classified as at risk of suboptimal surgical outcomes. This pattern of answers was however shown to have the best predictive capabilities when developing the algorithm with recursive partitioning. This situation underlines the concept that a predictor is not necessarily a determinant.

### Strengths of the study

The developed PA is, to our knowledge, the first one of its kind to discriminate THA or HR results based on more than one parameter, namely patients’ functional, stiffness and pain levels as well as their perception of the replaced hip joint. In the context of a lack of an accepted standard of surgical failure, this approach increases the likelihood of the patients thusly classified to truly present subpar outcomes. Moreover, this classification identified 15.1 % of the patients as having unsuccessful outcomes, well in line with the published proportions of what can be considered a suboptimal outcome [8]. Finally, the rigorous statistical analysis employed in the development of the PA underlines the stringency of our approach.

### Limitations of the study

Due to its retrospective design, the study has a certain risk of selection bias. Compared to the subjects excluded due to missing data, the included participants were, on average, younger, had a greater number of comorbidities and a greater proportion were male (*p <* 0.05, data not shown). Nevertheless, the preoperative baseline status as measured by the WOMAC domains as well as the total WOMAC score was not statistically significantly different between the included and the non-included subjects (*p >* 0.05, data not shown). Additionally, only 265 out of the 1207 procedures (22.0 %) that were performed during the study time period met the inclusion criteria, therefore potentially limiting the generalizability of the results. Moreover, the population under study was patients undergoing primary unilateral hip replacement procedures, which precludes the utilization of this tool for patients undergoing revision or bilateral interventions. Recent evidence identifies other variables potentially associated with hip arthroplasty outcomes that were not included in our study, thus potentially limiting the pool of candidate predictor variables. Although there is no consensus regarding the optimal sample size for developing models employing recursive partitioning, the progressively smaller number of cases in the leafs as the tree was built may limit the reliability of the findings, prompting further development in a subsequent study. Before employing it in a clinical setting, the decision rule has to be validated in a different sample of patients. Moreover, the performance of the PA has to be compared to clinical judgement alone and its financial impacts require evaluation.

## Conclusions

The developed PA may discriminate with excellent capabilities the patients undergoing hip arthroplasty that are at the highest risk of suboptimal pain, functional limitations and joint perception outcomes on an average of 15 months following the intervention. Its implementation has the potential of targeting susceptible individuals such as to modify their risk profile, and eventually, improve surgical results.

## Additional files

Additional file 1:
**STROBE Statement Checklist of items that should be included in reports of** ***cohort studies***. (PDF 95 kb) Additional file 2:
**Appendix contains six prediction algorithms that were also considered, along with their respective two by two tables and validity measures.** (DOCX 1064 kb)

## Abbreviations

BMI: Body mass index
CI: Confidence interval
HR: Resurfacing hip arthroplasty
OA: Osteoarthritis
PA: Prediction algorithm
THA: Total hip arthroplasty
STROBE: Strengthening the Reporting of Observational Studies in Epidemiology
WOMAC: Western Ontario and McMaster Universities Osteoarthritis Index

## References

[1] Zhang W, Doherty M, Arden N, Bannwarth B, Bijlsma J, Gunther KP (2005). EULAR evidence based recommendations for the management of hip osteoarthritis: report of a task force of the EULAR Standing Committee for International Clinical Studies Including Therapeutics (ESCISIT). Ann Rheum Dis.

[2] Zhang W, Moskowitz R, Nuki G, Abramson S, Altman R, Arden N (2008). OARSI recommendations for the management of hip and knee osteoarthritis, Part II: OARSI evidence-based, expert consensus guidelines. Osteoarthritis Cartilage.

[3] Beaulé PE, Antoniades J (2005). Patient selection and surgical technique for surface arthroplasty of the hip. Orthop Clin North Am.

[4] Ethgen O, Bruyere O, Richy F, Dardennes C, Reginster JY (2004). Health-related quality of life in total hip and total knee arthroplastyA qualitative and systematic review of the literature. J Bone Joint Surg.

[5] Garbuz DS, Tanzer M, Greidanus NV, Masri BA, Duncan CP (2010). The John Charnley Award: Metal-on-metal hip resurfacing versus large-diameter head metal-on-metal total hip arthroplasty: a randomized clinical trial. Clin Orthop Relat Res.

[6] Girard J, Vendittoli P, Roy A, Lavigne M (2008). [Femoral offset restauration and clinical function after total hip arthroplasty and surface replacement of the hip: a randomized study]. Rev Chir Orthop Reparatrice Appar Mot.

[7] Vissers MM, Bussmann JB, Verhaar JA, Arends LR, Furlan AD, Reijman M (2011). Recovery of physical functioning after total hip arthroplasty: systematic review and meta-analysis of the literature. Phys Ther.

[8] Beswick AD, Wylde V, Gooberman-Hill R, Blom A, Dieppe P. What proportion of patients report long-term pain after total hip or knee replacement for osteoarthritis? A systematic review of prospective studies in unselected patients. BMJ Open 2012; doi:10.1136/bmjopen-2011-000435 10.1136/bmjopen-2011-000435 PMC3289991 22357571

[9] Anakwe RE, Jenkins PJ, Moran M (2011). Predicting dissatisfaction after total hip arthroplasty: a study of 850 patients. J Arthroplasty.

[10] Jones CA, Voaklander DC, Johnston DW, Suarez-Almazor ME (2000). Health related quality of life outcomes after total hip and knee arthroplasties in a community based population. J Rheumatol.

[11] Shimmin A, Baré J (2011). Comparison of functional results of hip resurfacing and total hip replacement: a review of the literature. Orthop Clin N Am.

[12] Vendittoli P, Rivière C, Roy A, Barry J, Lusignan D, Lavigne M (2013). Metal-on-metal hip resurfacing compared with 28-mm diameter metal-on-metal total hip replacement A randomised study with six to nine years’ follow-up. Bone Joint J.

[13] Caracciolo B, Giaquinto S (2005). Determinants of the subjective functional outcome of total joint arthroplasty. Archiv Gerentol Geriatr.

[14] Fortin PR, Clarke AE, Joseph L, Liang MH, Tanzer M, Ferland D (1999). Outcomes of total hip and knee replacement: preoperative functional status predicts outcomes at six months after surgery. Arthritis Rheum.

[15] Fortin PR, Penrod JR, Clarke AE, St‐Pierre Y, Joseph L, Bélisle P (2002). Timing of total joint replacement affects clinical outcomes among patients with osteoarthritis of the hip or knee. Arthritis Rheum.

[16] Gandhi R, Razak F, Davey JR, Mahomed NN (2010). Metabolic syndrome and the functional outcomes of hip and knee arthroplasty. J Rheumatol.

[17] Garbuz DS, Xu M, Duncan CP, Masri BA, Sobolev B (2006). Delays worsen quality of life outcome of primary total hip arthroplasty. Clin Orthop.

[18] Jones CA, Voaklander DC, Johnston DW, Suarez-Almazor ME (2001). The effect of age on pain, function, and quality of life after total hip and knee arthroplasty. Arch Intern Med.

[19] Judge A, Batra RN, Thomas GE, Beard D, Javaid MK, Murray DW (2014). Body mass index is not a clinically meaningful predictor of patient reported outcomes of primary hip replacement surgery: Prospective cohort study. Osteoarthritis Cartilage.

[20] Quintana JM, Escobar A, Aguirre U, Lafuente I, Arenaza JC (2009). Predictors of health-related quality-of-life change after total hip arthroplasty. Clin Orthop.

[21] Ramaesh R, Jenkins P, Lane JV, Knight S, Macdonald D, Howie C (2014). Personality, function and satisfaction in patients undergoing total hip or knee replacement. J Orthop Sci.

[22] Stevens M, Paans N, Wagenmakers R, van Beveren J, van Raay JJ, van der Meer K (2012). The influence of overweight/obesity on patient-perceived physical functioning and health-related quality of life after primary total hip arthroplasty. Obes Surg.

[23] Bellamy N, Buchanan W (1986). A preliminary evaluation of the dimensionality and clinical importance of pain and disability in osteoarthritis of the hip and knee. Clin Rheumatol.

[24] Bellamy N (1988). Validation study of WOMAC: a health status instrument for measuring clinically-important patient-relevant outcomes following total hip or knee arthroplasty in osteoarthritis. J Orthop Rheumatol.

[25] McConnell S, Kolopack P, Davis AM (2001). The Western Ontario and McMaster Universities Osteoarthritis Index (WOMAC): a review of its utility and measurement properties. Arthritis Care Res.

[26] Collins M, Lavigne M, Girard J, Vendittoli P-A (2012). Joint perception after hip or knee replacement surgery. Orthop Traumatol Surg Res.

[27] Breinman LFJ, Olshen R, Stome C (1984). Classification and regression trees.

[28] Strobl C, Boulesteix A-L, Augustin T (2007). Unbiased split selection for classification trees based on the Gini index. Comput Stat Data An.

[29] Simel DL, Samsa GP, Matchar DB (1991). Likelihood ratios with confidence: sample size estimation for diagnostic test studies. J Clin Epidemiol.

[30] Efron BTR (1993). An introduction to the bootstrap.

[31] Slaven EJ (2012). Prediction of functional outcome at six months following total hip arthroplasty. Phys Ther.

[32] Stiell IG, Greenberg GH, McKnight RD, Nair RC, McDowell I, Worthington JR (1992). A study to develop clinical decision rules for the use of radiography in acute ankle injuries. Ann Emerg Med.

[33] Stiell IGGG, Wells GA, McKnight RD, Cwinn AA, Cacciotti T, McDowell I (1995). Derivation of a decision rule for the use of radiography in acute knee injuries. Ann Emerg Med.

[34] Braeken AM, Lochhaas-Gerlach JA, Gollish JD, Myles JD, Mackenzie TA (1997). Determinants of 6–12 month postoperative functional status and pain after elective total hip replacement. Int J Qual Health Care.

[35] Kessler S, Kafer W (2007). Overweight and obesity: two predictors for worse early outcome in total hip replacement?. Obesity.

[36] Kleinbaum DG (1988). Applied regression analysis and multivariable methods. CengageBrain.

