# Supplementary material for: Identification of patients with suboptimal results after hip arthroplasty: development of a preliminary prediction algorithm
Source: BMC Musculoskelet Disord. 2015 Oct 5;16:279. doi: 10.1186/s12891-015-0720-1 (PMC4595123; doi:10.1186/s12891-015-0720-1)
Supplement: Additional file 2: — Appendix contains six prediction algorithms that were also considered, along with their respective two by two tables and validity measures. (DOCX 1064 kb) [file 12891_2015_720_MOESM2_ESM.docx]

**Figure 1** Graphical representation of prediction algorithm (PA) 1


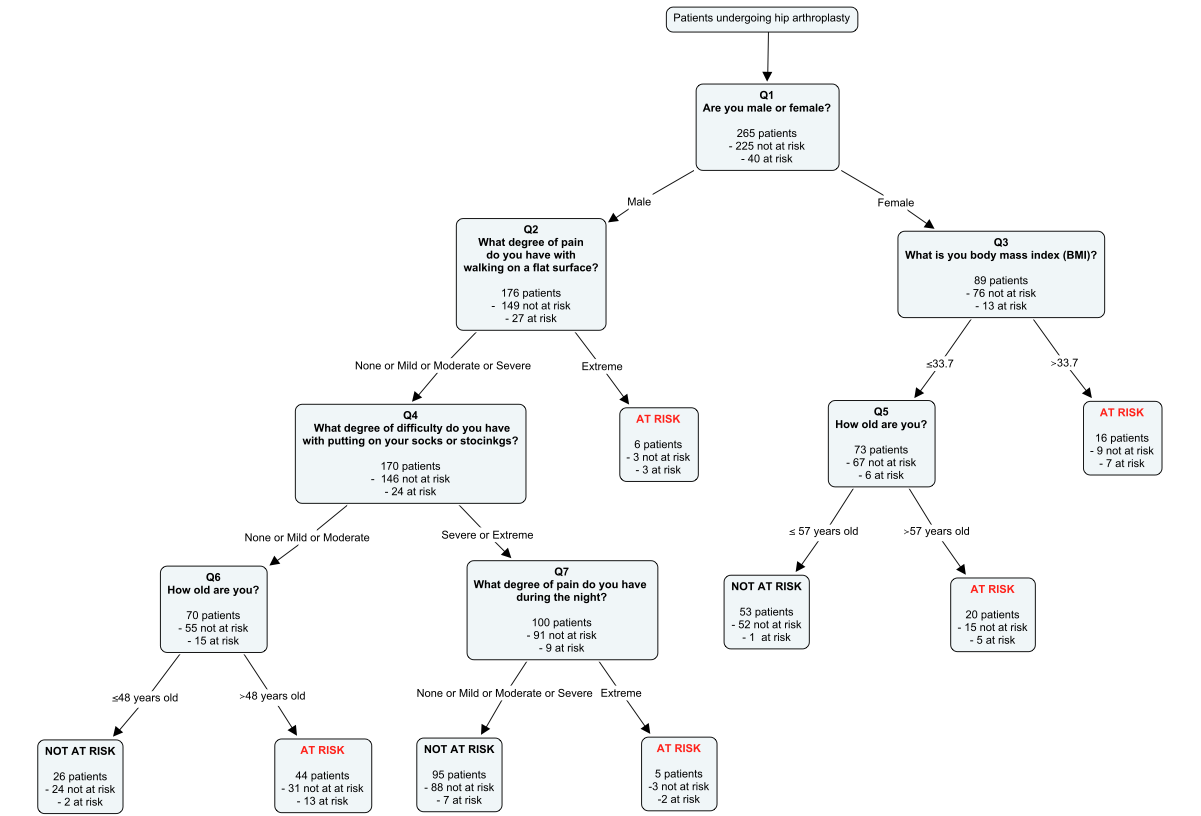


**Table 1.1** Two by Two table of predicted versus actual outcomes of the PA1

|  | **Actual Outcome** | |
| --- | --- | --- |
| **Predicted outcome** | **AT RISK** | **NOT AT RISK** |
|  | Worst postoperative WOMAC ~~tertile~~ quartile (>11.5/100) & “Artificial with minimal or major limitations” joint perception | Postoperative WOMAC ≤ 11.5/100 or ‘’Artificial with no limitations’’ or ‘’Natural joint’’ joint perception |
| **AT RISK** | 30 | 61 |
| **NOT AT RISK** | 10 | 164 |
| *TOTAL* | *40* | *225* |

**Table 1.2** Validity measures of the PA1

| **Measure** | **Estimates in training sample** |
| --- | --- |
| ***Sensitivity % (95% CI)*** | 75.0 (59.8-85.8) |
| ***Specificity % (95% CI)*** | 72.9 (66.7-78.3) |
| ***Positive predictive value % (95% CI)*** | 33.0 (24.2-43.1) |
| ***Negative predictive value % (95% CI)*** | 94.3 (89.7-96.8) |
| ***Positive likelihood ratio (95% CI)*** | 2.77 (2.09-3.66) |
| ***Negative likelihood ratio (95% CI)*** | 0.34 (0.20-0.59) |

**Figure 2** Graphical representation of PA2


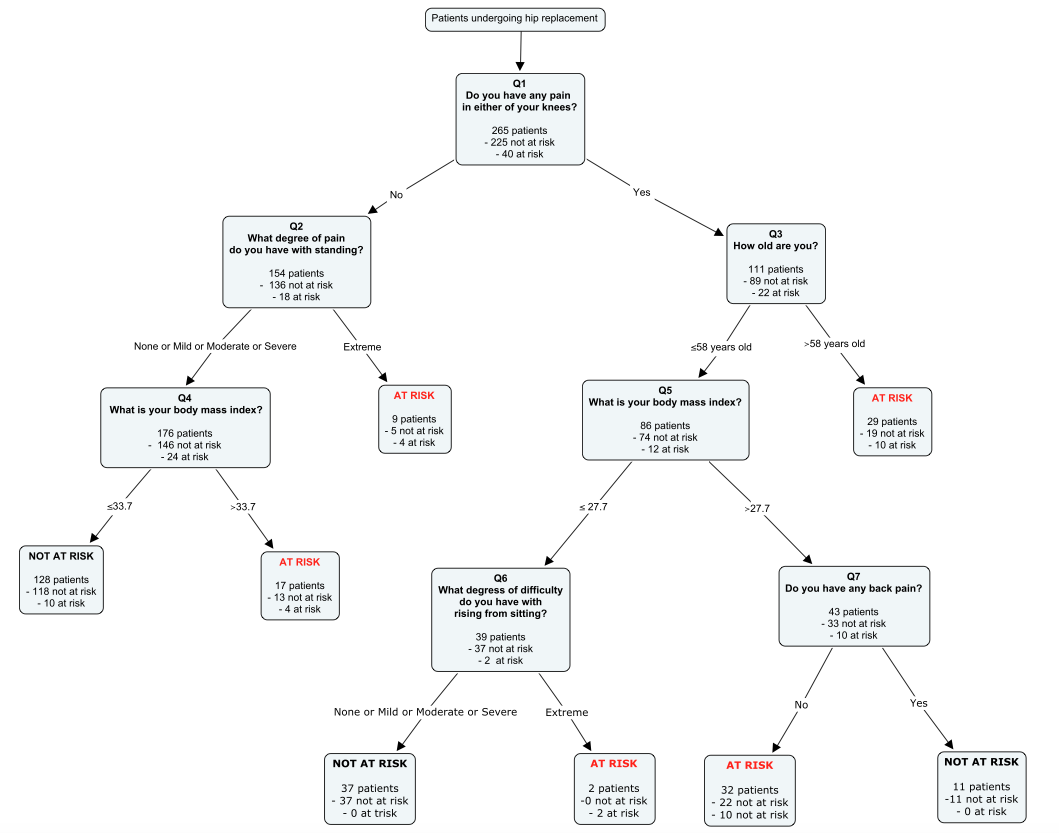


**Table 2.1** Two by Two table of predicted versus actual outcomes of the PA2

|  | **Actual Outcome** | |
| --- | --- | --- |
| **Predicted outcome** | **AT RISK** | **NOT AT RISK** |
|  | Worst postoperative WOMAC ~~tertile~~ quartile (>11.5/100) & “Artificial with minimal or major limitations” joint perception | Postoperative WOMAC ≤ 11.5/100 or ‘’Artificial with no limitations’’ or ‘’Natural joint’’ joint perception |
| **AT RISK** | 30 | 59 |
| **NOT AT RISK** | 10 | 166 |
| *TOTAL* | *40* | *225* |

**Table 1.2** Validity measures of the PA2

| **Measure** | **Estimates in training sample** |
| --- | --- |
| ***Sensitivity % (95% CI)*** | 75.0 (59.8-85.8) |
| ***Specificity % (95% CI)*** | 73.8 (67.7-79.1) |
| ***Positive predictive value % (95% CI)*** | 33.7 (24.7-44.0) |
| ***Negative predictive value % (95% CI)*** | 94.3 (89.9-96.9) |
| ***Positive likelihood ratio (95% CI)*** | 2.86 (2.16-3.80) |
| ***Negative likelihood ratio (95% CI)*** | 0.34 (0.20-0.58) |

**Figure 3** Graphical representation of PA3


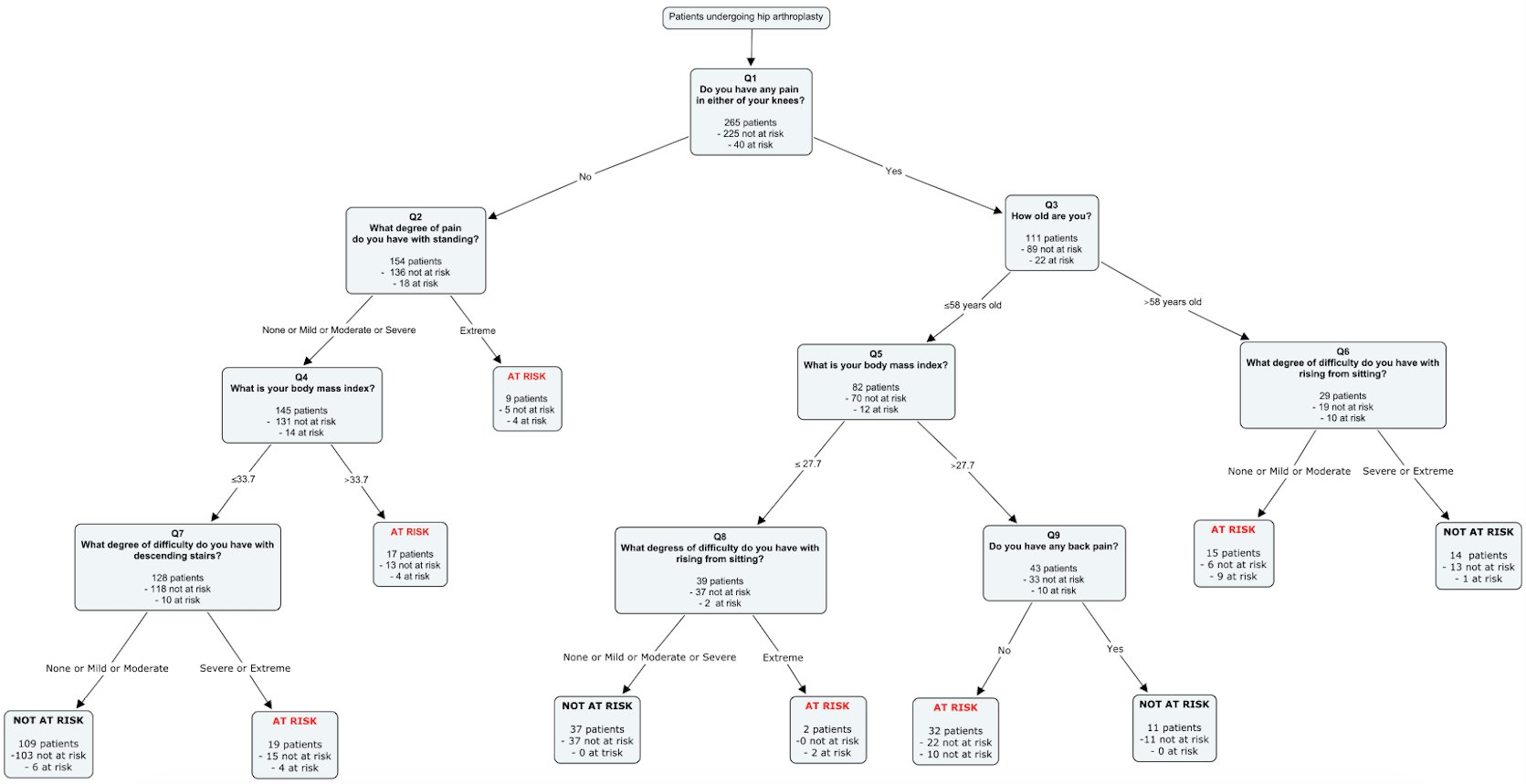


**Table 3.1** Two by Two table of predicted versus actual outcomes of the PA3

|  | **Actual Outcome** | |
| --- | --- | --- |
| **Predicted outcome** | **AT RISK** | **NOT AT RISK** |
|  | Worst postoperative WOMAC ~~tertile~~ quartile (>11.5/100) & “Artificial with minimal or major limitations” joint perception | Postoperative WOMAC ≤ 11.5/100 or ‘’Artificial with no limitations’’ or ‘’Natural joint’’ joint perception |
| **AT RISK** | 33 | 61 |
| **NOT AT RISK** | 7 | 164 |
| *TOTAL* | *40* | *225* |

**Table 3.2** Validity measures of the PA3

| **Measure** | **Estimates in training sample** |
| --- | --- |
| ***Sensitivity % (95% CI)*** | 82.5 (68.1-91.3) |
| ***Specificity % (95% CI)*** | 72.9 (66.7-78.3) |
| ***Positive predictive value % (95% CI)*** | 35.1 (26.2-45.2) |
| ***Negative predictive value % (95% CI)*** | 95.9 (91.8-98.0) |
| ***Positive likelihood ratio (95% CI)*** | 3.04 (2.35-3.94) |
| ***Negative likelihood ratio (95% CI)*** | 0.24 (0.12-0.47) |

**Figure 4** Graphical representation of PA4


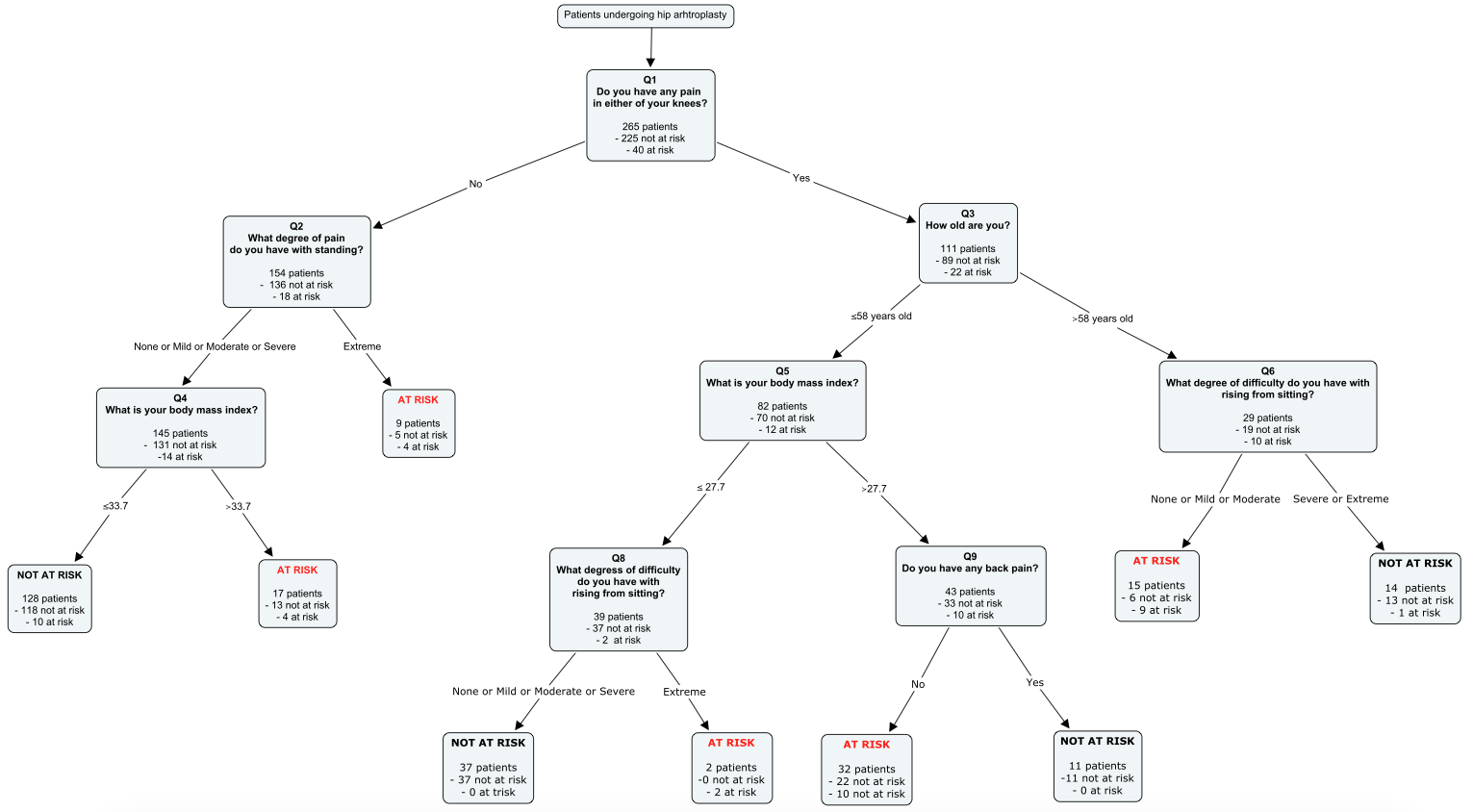


**Table 4.1** Two by Two table of predicted versus actual outcomes of the PA4

|  | **Actual Outcome** | |
| --- | --- | --- |
| **Predicted outcome** | **AT RISK** | **NOT AT RISK** |
|  | Worst postoperative WOMAC ~~tertile~~ quartile (>11.5/100) & “Artificial with minimal or major limitations” joint perception | Postoperative WOMAC ≤ 11.5/100 or ‘’Artificial with no limitations’’ or ‘’Natural joint’’ joint perception |
| **AT RISK** | 29 | 46 |
| **NOT AT RISK** | 11 | 179 |
| *TOTAL* | *40* | *225* |

**Table 4.2** Validity measures of the PA4

| **Measure** | **Estimates in training sample** |
| --- | --- |
| ***Sensitivity % (95% CI)*** | 72.5 (57.2-83.9) |
| ***Specificity % (95% CI)*** | 79.6 (73.8-84.3) |
| ***Positive predictive value % (95% CI)*** | 38.7 (28.5-50.0) |
| ***Negative predictive value % (95% CI)*** | 94.2 (89.9-96.7) |
| ***Positive likelihood ratio (95% CI)*** | 3.55 (2.57-4.89) |
| ***Negative likelihood ratio (95% CI)*** | 0.35 (0.21-0.57) |

**Figure 5** Graphical representation of PA5


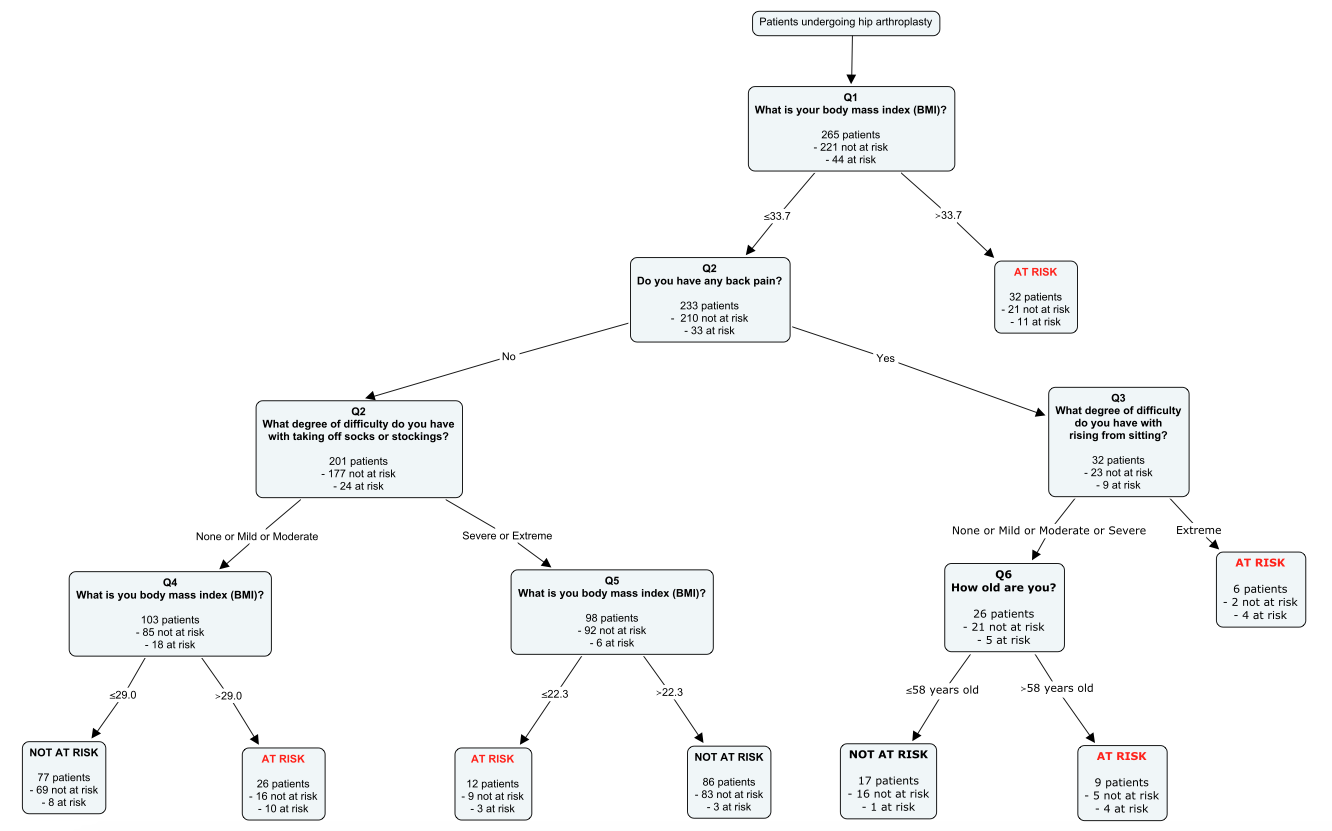


**Table 5.1** Two by Two table of predicted versus actual outcomes of the PA5

|  | **Actual Outcome** | |
| --- | --- | --- |
| **Predicted outcome** | **AT RISK** | **NOT AT RISK** |
|  | Worst postoperative WOMAC ~~quartile~~ tertile (>~~11.5~~ 9.4/100) & “Artificial with minimal or major limitations” joint perception | Postoperative WOMAC ≤ ~~11.5~~ 9.4/100 or ‘’Artificial with no limitations’’ or ‘’Natural joint’’ joint perception |
| **AT RISK** | 32 | 53 |
| **NOT AT RISK** | 12 | 168 |
| *TOTAL* | *44* | *221* |

**Table 5.2** Validity measures of the PA5

| **Measure** | **Estimates in training sample** |
| --- | --- |
| ***Sensitivity % (95% CI)*** | 72.7 (58.2-83.7) |
| ***Specificity % (95% CI)*** | 76.0 (70.0-81.2) |
| ***Positive predictive value % (95% CI)*** | 37.6 (28.1-48.3) |
| ***Negative predictive value % (95% CI)*** | 93.3 (88.7-96.1) |
| ***Positive likelihood ratio (95% CI)*** | 3.03 (2.26-4.08) |
| ***Negative likelihood ratio (95% CI)*** | 0.36 (0.22-0.59) |

**Figure 6** Graphical representation of PA6


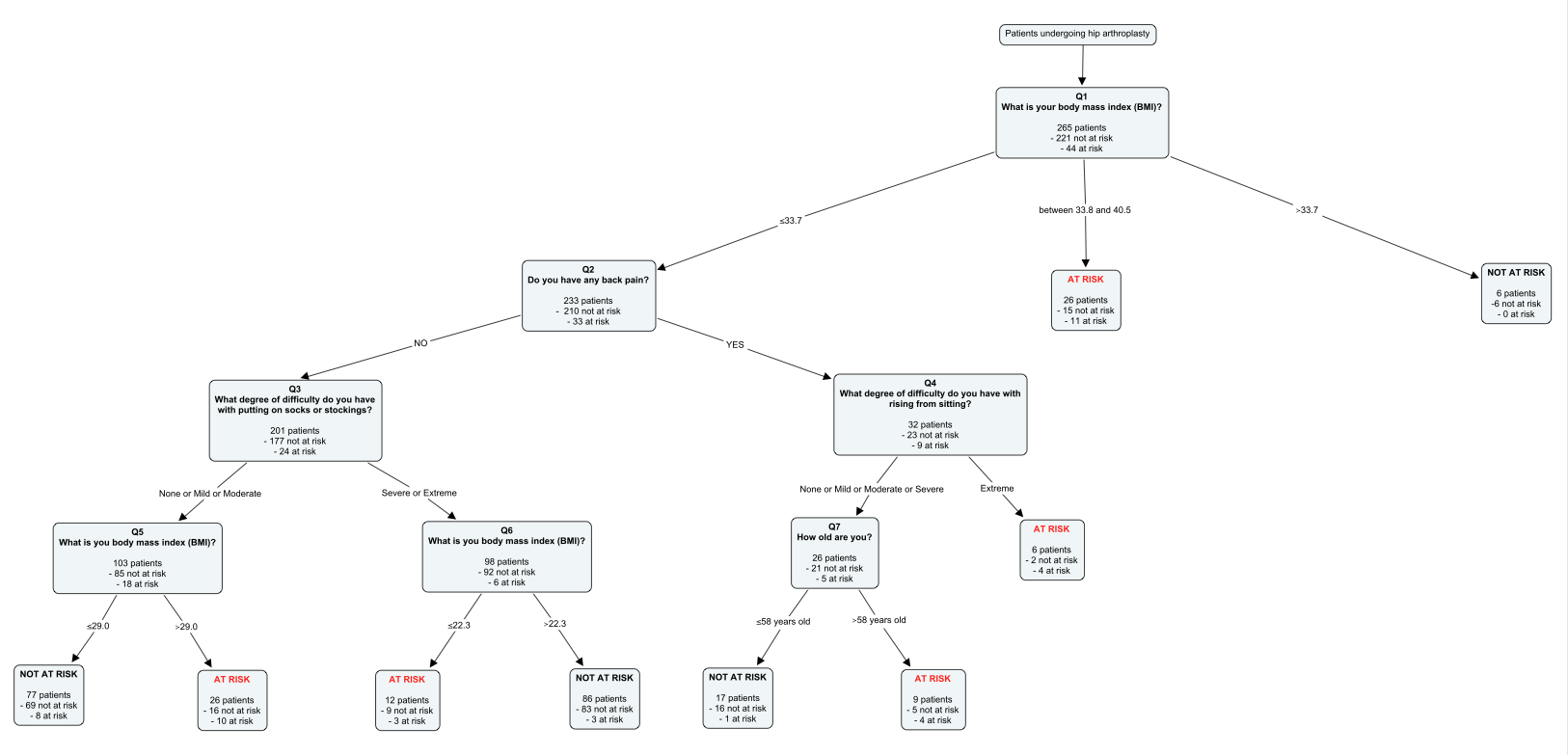


**Table 6.1** Two by Two table of predicted versus actual outcomes of the PA6

|  | **Actual Outcome** | |
| --- | --- | --- |
| **Predicted outcome** | **AT RISK** | **NOT AT RISK** |
|  | Worst postoperative WOMAC ~~quartile~~ tertile (>~~11.5~~ 9.4) & “Artificial with minimal or major limitations” joint perception | Postoperative WOMAC ≤ ~~11.5~~ 9.4/100 or ‘’Artificial with no limitations’’ or ‘’Natural joint’’ joint perception |
| **AT RISK** | 32 | 47 |
| **NOT AT RISK** | 12 | 174 |
| *TOTAL* | *44* | *221* |

**Table 6.2** Validity measures of the PA6

| **Measure** | **Estimates in training sample** |
| --- | --- |
| ***Sensitivity % (95% CI)*** | 72.7 (58.2-83.7) |
| ***Specificity % (95% CI)*** | 78.7 (72.9-83.6) |
| ***Positive predictive value % (95% CI)*** | 40.5 (30.4-51.5) |
| ***Negative predictive value % (95% CI)*** | 93.5 (89.1-96.3) |
| ***Positive likelihood ratio (95% CI)*** | 3.42 (2.50-4.67) |
| ***Negative likelihood ratio (95% CI)*** | 0.35 (0.21-0.56) |
